# Intraoperative Molecular Imaging With Pafolacianine in Resection of Occult Pulmonary Malignancy in the ELUCIDATE Trial

**DOI:** 10.1016/j.athoracsur.2024.10.001

**Published:** 2024-10-21

**Authors:** David Rice, Sunil Singhal, Emma Niemeyer, Inderpal Sarkaria, Linda W. Martin, Michael I. Ebright, Brian E. Louie, Tommy Lee, Jarrod D. Predina

**Affiliations:** 1 MD Anderson Cancer Center, Houston, Texas; 2 Center for Precision Surgery, Perelman School of Medicine at the University of Pennsylvania, Philadelphia, Pennsylvania; 3 Division of Thoracic Surgery, Department of Surgery, Perelman School of Medicine at the University of Pennsylvania, Philadelphia, Pennsylvania; 4 University of Texas Southwestern Medical Center, Dallas, Texas; 5 Section of Thoracic Surgery, Department of Surgery, University of Virginia School of Medicine, Charlottesville, Virginia; 6 Columbia University Irving Medical Center, New York, New York; 7 Swedish Cancer Institute and Medical Center, Seattle, Washington; 8 On Target Laboratories, Inc, West Lafayette, Indiana

## Abstract

**BACKGROUND:**

Clinical studies have demonstrated that intraoperative molecular imaging (IMI) with pafolacianine identifies occult pulmonary lesions that are not identified by preoperative computed tomography or by intraoperative inspection techniques in ~20% of patients. This study describes occult lesion clinical data and evaluates characteristics so that surgeons can better incorporate this emerging technology into clinical decision making.

**METHODS:**

Participants (n = 100) enrolled in a phase 3 trial of IMI with pafolacianine during pulmonary resection (Enabling Lung Cancer Identification Using Folate Receptor Targeting [ELUCIDATE]; NCT04241315) were identified. Participants underwent preoperative computed tomography with 1.25-mm slices. Patient and lesion characteristics were analyzed. Positive predictive value and false positive rates were tabulated for IMI fluorescent lesions, with predictors of malignant vs benign occult lesions described.

**RESULTS:**

IMI identified 29 occult lesions in 23 (23%) participants. Seventeen of 29 (58%) lesions were identified within the same lobe as known lesions; 12 of 29 (42%) were identified in a different lobe from the suspicious nodule known by preoperative assessment. Twenty-three of 29 (79%) of occult lesions found by IMI were resected with an additional wedge resection. Ten of 29 (34%) lesions identified by IMI were malignant. There was no additional morbidity in participants with lesions resected. With pafolacianine, 7 participants had a synchronous primary stage I lung cancer identified, and 1 participant had additional metastases identified.

**CONCLUSIONS:**

IMI with pafolacianine identifies occult malignant lesions during pulmonary resection despite thorough preoperative imaging and intraoperative assessment by experienced surgeons.

Intraoperative molecular imaging (IMI) is an emerging technique that involves systemic delivery of targeted, optical contrast agents that selectively accumulate in tumors and result in intraoperative tumor fluorescence.^1^ This technique has been developed to improve intraoperative decision making by providing real-time, visual feedback to the surgeon.^1–5^ In 2022, pafolacianine (On Target Laboratories) received approval from the U.S. Food and Drug Administration as an adjunct for intraoperative identification of pulmonary lesions, thereby extending the original indication for ovarian lesions initially approved in 2021. Specifically for lung nodules, a multicenter phase 3 randomized trial (Enabling Lung Cancer Identification Using Folate Receptor Targeting [ELUCIDATE]; NCT04241315) demonstrated that pafolacianine, in combination with standard of care minimally invasive pulmonary resection (video-assisted thoracoscopic surgery and robotic-assisted thoracoscopic surgery), affected 53% of participants randomized to IMI.^6^ These clinically significant events (CSEs) included identification of inadequate margins (38% of participants), improvements of lesion localization (19%), and discovery of otherwise occult synchronous malignant lesions (8%). Of note, 10% of participants had more than 1 CSE in this phase 3 trial cohort. These findings validated results found in an earlier nonrandomized, multicenter phase 2 trial of pafolacianine (OTL38 Injection for Intraoperative Imaging of Folate Receptor Positive Lung Nodules; NCT02872701) involving a similar cohort of participants in which 26% of participants with non-small cell lung cancer (NSCLC) experienced CSEs.^7^

Although the impact of rapid identification of positive margins and reliable localization of subtle subsolid or ground-glass opacities is fairly obvious, the benefits of discovering radiographically occult, synchronous cancers are less clear without long-term data. One could make the argument that resection of such lesions could result in overtreatment. For example, it is fairly common practice for thoracic surgeons to treat only evolving lesions in patients with multifocal ground-glass opacities ^8,9^ or in certain scenarios associated with diffuse idiopathic pulmonary neuroendocrine cell hyperplasia.^10^ These approaches appear to minimize morbidity, maximize parenchymal preservation, and have little impact on overall survival. On the other end of this debate, removal of additional lesions may represent proper execution of one the primary tenants of oncologic surgery, which is to achieve “complete local control.”^1^

Survival and recurrence data were beyond the study goals of the phase 2 and phase 3 protocols, which evaluated IMI with pafolacianine as a real-time targeted visualization tool, and were therefore not collected. Additionally, retrospective collection would be of limited value because median postsurgical follow-up is currently less than 2 years.

With recent Food and Drug Administration approval and the seemingly rapid implementation of this technology, the goal of this report is to summarize and further analyze data from the ELUCIDATE trial that pertain to intraoperative discovery of occult lesions. We seek to provide granularity regarding occult lesion location, size, histology, and test characteristics (positive predictive value). In addition, we seek to understand which clinical variables may predict malignancy in lesions identified solely with IMI. This information will be critical for thoracic surgeons using pafolacianine and will allow for improved incorporation of IMI into established intraoperative decision-making algorithms.

## MATERIAL AND METHODS

### DATA SOURCE.

The data for this report were derived from the original ELUCIDATE trial database and listings generated from iMedidata Rave software version 2021.1.3 (Medidata Solutions).

### STUDY DESIGN.

This is a post hoc analysis involving prospectively collected data from ELUCIDATE, which was a multicenter, randomized phase 3 trial aimed at evaluating the clinical utility of IMI with pafolacianine during pulmonary resection.^6^ The trial protocol was approved by an Institutional Review Board (November 25, 2019, # Pro00040361). The study was approved by the Institutional Review Board at each enrolling center as part of the original clinical trial agreements. The full protocol can be found in the trial report.^6^ Written informed consent, including consent for data publication, was obtained from each participant before initiating screening procedures. Briefly, participants receiving pafolacianine and undergoing IMI were identified from the ELUCIDATE database. Participants received 0.025 mg/kg of pafolacianine delivered intravenously over 60 minutes within 1 to 24 hours before surgery and IMI. All participants underwent a high-resolution computed tomographic (CT) scan with at least 1.25-mm axial cuts before enrollment. Otherwise, occult lesions identified exclusively by IMI were tallied. Occult lesions were defined as nodules that were not identified on preoperative imaging or by traditional intraoperative techniques, such as visual inspection or palpation techniques. All preoperative imaging was reviewed by institutional radiologists and underwent independent central review by a qualified thoracic surgeon. Variables that could possibly predict malignancy in occult lesions were incorporated into multivariable models and included age at the time of resection, sex, preoperatively known tumor location, preoperatively known tumor size, clinical stage, occult lesion size, occult lesion location, pathologic stage, and history of previous cancers.

### STATISTICAL ANALYSIS.

This is primarily a descriptive report with data expressed in median values with interquartile ranges or percentages, unless otherwise noted. Positive predictive value and false positive rate were tabulated for lesions based on fluorescence alone. Finally, predictors of malignant occult lesions (true positives) versus benign occult lesions (false positives) were examined. Statistical analyses were performed using Sigma Plot for Windows Version 15.

## RESULTS

## PARTICIPANT CHARACTERISTICS.

A total of 100 participants who received pafolacianine in ELUCIDATE and were randomized to near-infrared (NIR) imaging were included. A comprehensive summary of participant and primary lesion characteristics is provided in Table 1. Most participants had stage I or II NSCLC (n = 64), with a median primary tumor size of 13 mm in greatest dimension. The next most common diagnosis was metastatic disease to the lungs (n = 21). Preoperative CT scans were completed at an average of 6.77 weeks (range, 2–15 weeks) before surgery.

### OCCULT LESION CHARACTERISTICS.

In 23 participants, 29 occult lesions were resected after being identified exclusively by IMI (Figure). Twenty-one participants had a single additional lesion identified and resected, 1 participant had 2 additional lesions, and 1 participant had 4 lesions (Tables 2, 3). Most occult lesions were less than 1 cm (n = 15; 53%); however, they ranged in size from 0.2 cm to 2.0 cm (median, 0.5 cm). Those resected lesions that contained no identifiable disease process on final pathologic review were not measured (Table 3).

Seventeen (58%) of those 29 occult lesions identified solely with IMI were within the same lobe as primary nodules identified by preoperative CT scans, and 12 (42%) were found in a different lobe (Table 2). These otherwise occult lesions were most commonly resected with an additional wedge resection (23 of 29 lesions; 79%). The remaining (6 of 29 occult lesions; 21%) were included in a single resection, which also included the known lung lesion (5 lobectomies, 1 segmentectomy) (Table 3). On review of the investigator questionnaire, there was no clear perioperative morbidity associated with resection of occult lesions and no increase in pulmonary complications, as determined by safety event data review.

On histopathologic review of the 29 lesions identified by IMI, 7 (24%) were invasive pulmonary adenocarcinoma, 3 (10%) were metastases from other primary sites, 1 (3%) was adenocarcinoma in situ, and 18 (62%) were benign lesions (Table 3). Of the 7 occult NSCLCs identified, 4 measured greater than 1.0 cm (1.0 cm, 1.1 cm, 1.8 cm, and 2.0 cm), and each of these lesions had predominantly lepidic histologic features. Of the 18 benign lesions, 15 (83%) were benign tissue, 2 (11%) were granulomas, and 1 (6%) was organizing pneumonia.

Four of 8 participants with malignant occult lesions found by IMI had lesions located in a different lobe, and all these lesions were removed by wedge resection (Table 2). The other 4 participants had occult malignant lesions within the same lobe as the known tumor; 2 lesions were managed with wedge resection, and 2 lesions were included during lobectomy.

An independent central review of the preoperative CT scan was completed for participants with a malignant occult lesion. Of the 10 malignant lesions discovered with pafolacianine, none were found in retrospective review by the independent reviewer and further were not identified in the original radiology report and by the surgeon review of the scans.

### TEST CHARACTERISTICS.

The true positive rate of fluorescence predicting malignancy (invasive NSCLC or metastases from a distant primary tumor) was found to be 37%, with 1 lesion identified as true positive for adenocarcinoma in situ. The positive predictive value was 38% of malignancy in occult lesions identified with IMI. True negative and false negative rates could not be calculated because otherwise healthy tissue was not resected for purposes of establishing a negative control.

### PREDICTORS OF OCCULT MALIGNANCY.

Univariate analysis was performed to understand better which participant and primary lesion variables predicted malignancy in occult lesions identified by IMI. We found no significant relationships between any pair of variables analyzed (Table 4).

## COMMENT

IMI is an emerging technique that provides real-time, visual information to improve intraoperative decision making by the surgeon. In this report, we expand on the previously described ELUCIDATE trial, which is a stage 3 multicenter trial involving IMI with the folate receptor–targeted agent, pafolacianine. We provide further analysis pertaining to those additional, nontarget lesions identified exclusively by IMI, and not by traditional preoperative and intraoperative techniques—“occult lesions.” The goal of this report is to provide data so that thoracic surgeons can understand the clinical relevance of such fluorescent lesions more clearly and, in turn, can better incorporate IMI data into established decision-making algorithms and clinical acumen.

Previous data from clinical experiences with pafolacianine-based IMI for NSCLC suggested that 26% to 53% of patients derive clinical benefits.^6,7^ These benefits have fallen into 3 general categories marked by improvements in the following: (1) localization of small, subpleural, or subsolid lesions; (2) detection of close or positive margins during sublobar resection; and (3) identification of otherwise occult malignant lesions at the time of resection.

In this report, we reexamine the ELUCIDATE cohort and further describe data on occult lesions that were identified exclusively by IMI. In this cohort of 100 participants, 23 participants were found to have additional lesions. Twentynine lesions were found in total, 10 (34%) of which represented additional malignancies. Seven lesions were considered synchronous primary lung cancers (adenocarcinoma), and 3 were metastatic lesions from a patient with a history of a chordoma. Occult lesions were most frequently initially managed with wedge resection (26 of 29), which was found to add negligible morbidity on review of trial questionnaires and analysis of safety data. For incidentally identified NSCLC in the same lobe as the preoperatively known lesion (T3 lesions), surgeons participating in ELUCIDATE used lobectomy (n = 2) or wedge resection (n = 2). For NSCLC in a different lobe (n = 4) than that of preoperatively known lesions (T4 lesions), the additional lesion was managed by sublobar resection (Table 3). Although this was the observed trend, we recognize that management of occult *malignant* lesions identified by IMI will be nuanced and patient-specific. In those circumstances in which occult lesions were metastases originating from distant sites or benign lesions, wedge resection was typically performed. Unfortunately, we could not identify predictors of malignancy in our univariate analysis, likely because the initial trial design of ELUCIDATE was not powered for such analyses. Given the lack of predictors of malignancy in occult lesions, surgeons identifying occult lesions will need to make management decisions on the basis of clinical risk factors and frozen section analysis.

The finding of additional malignant occult lesions in ELUCIDATE in 8% of patients was similar to the experience observed in the phase 1 trial,^11^ which found occult lesions in 10% of patients, and was closely matched to the multicenter phase 2 trial,^7^ which found malignant occult lesions in 8% of patients. Furthermore, a recent “real-world” experience from the University of Pittsburgh Medical Center (Pittsburgh, PA) reported that malignant occult lesions were identified in 8% of patients.^12^ The rate of detection for occult malignant lesions in ELUCIDATE and in other experiences reported with pafolacianine falls within the frequency of occult lesion rates of 8% to 9% reported in the literature.^13^

Although IMI with pafolacianine indeed does identify otherwise occult malignancies, it is important to acknowledge that the clinical impact of resection remains uncertain. Previous human experiences involving IMI are limited. For example, phase 1 and phase 2 trials were non-randomized experiences focused on feasibility, and it is therefore difficult to draw objective conclusions on the basis of improvements in overall or disease-free survivorship.^1,4,7^ With regard to the randomized phase 3 trial (ELUCIDATE), the median follow-up period is only 2 years.^6^ In the absence of these critical data, it is unclear whether resection of additional lesions is of oncologic benefit, particularly in the case of identifying synchronous NSCLC. Interestingly, there is a recent report involving indocyanine green, a similar, but nonspecific, NIR imaging agent, which suggests that resection of additional lesions improves disease-free survival during metastasectomy.^14^

In terms of practicality, IMI with pafolacianine requires only minor modifications to standard workflow. In most scenarios, pafolacianine was delivered preoperatively in the holding area, with 95% of patients completing the infusion. Toxicity was minimal, as previously noted.^6^ Intraoperatively, molecular imaging required a median time of 2 minutes (maximum, 23 minutes). During traditional video-assisted thoracoscopic surgery, most participating surgeons used an NIR camera, thus no additional ports were required. For those surgeons using robotic-assisted thoracoscopic surgery techniques, in general participating surgeons initially identified and marked lesions using the specialized NIR camera systems and then proceeded with robotic resection. Newer-generation cameras and robots will have the added ability to identify and detect pafolacianine.

Although IMI with pafolacianine provides a safe and practical approach to identify otherwise occult malignancies at the time of pulmonary resection, long-term benefits are unclear at this time. As such, we think it is important to emphasize that IMI was developed to provide *additional* data to the surgeon, and not to *replace* surgeon’s decision making. Intraoperative fluorescent feedback with IMI should serve as a supplement to traditional modalities such as preoperative imaging, intraoperative cues, and clinical variables. Potential benefits of resecting additional malignant lesions must be balanced with considerations of unnecessary parenchymal resection in circumstances of “false positives” and the extent of additional resection required. In addition, the surgeon should consider the clinical impact when the primary diagnosis is early-stage lung cancer vs pulmonary metastases. This report provides additional data so that the surgeon can better determine whether resection of additional lesions identified by IMI is warranted.

## Figures and Tables

**FIGURE:**
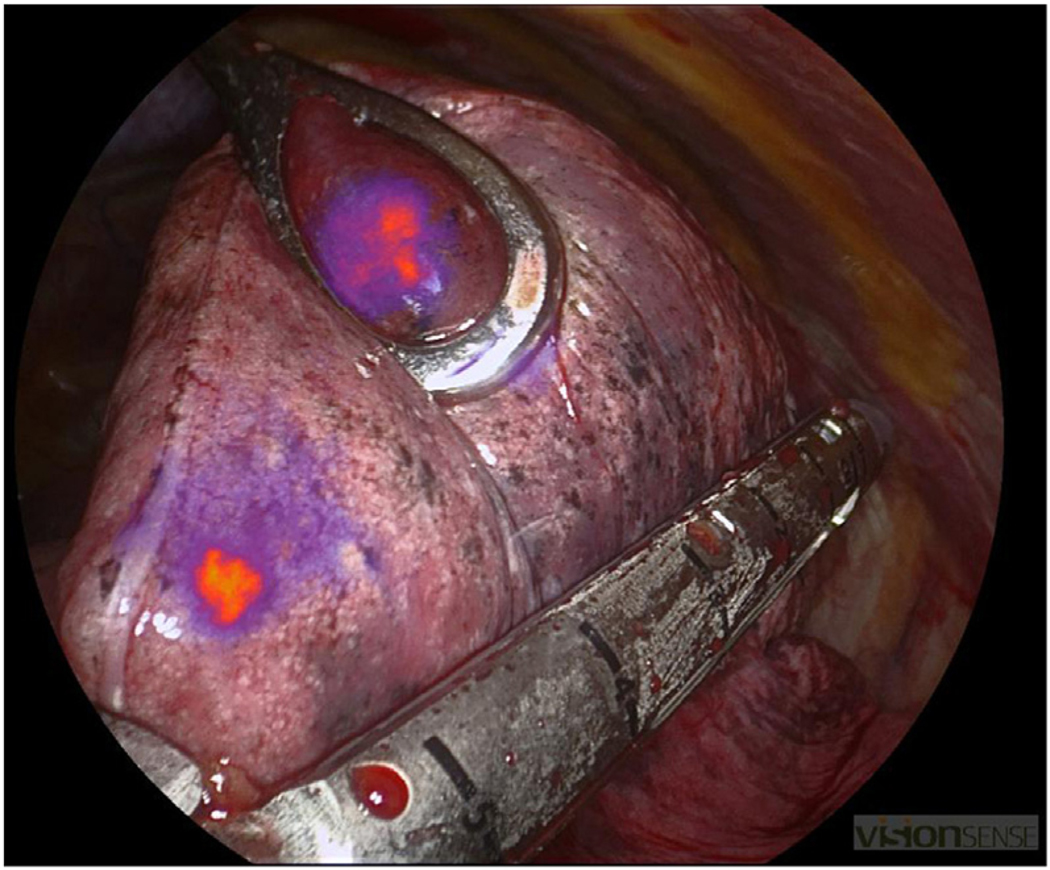
Intraoperative molecular imaging with pafolacianine. The primary nodule shown here was visualized under near-infrared light in the right upper lobe of the lung and measured 5.2 mm^3^ in greatest length.

**TABLE 1:** Characteristics of Phase 3 Participants From the ELUCIDATE Trial Who Received Pafolacianine and Underwent Intraoperative Molecular Imaging

Variables	n (%)

Sex	
Male	39 (39)
Female	61 (61)
Age, y	
≥65	64 (64)
<65	36 (36)
Preoperatively identified primary lesion size by CT, cm	
≥2.0	29 (29)
≥1 to <2.0	49 (49)
<1.0	21 (21)
Not measured	1 (1)
Histopathologic diagnosis of primary	
lesion	
NSCLC	64 (64)
Metastasis	21 (21)
Atypical carcinoid	1 (1)
Benign lesion	11 (11)
Other	3 (3)
Pack-year smoking history, y	
≥20	54 (76)
<20	17 (24)
Smoking status	
Active	19 (19)
Former	52 (52)
Never	29 (29)
Any cancer history other than lung	
Yes	50 (50)
No	50 (50)
Any lung cancer history	
Yes	22 (22)
No	78 (78)
Race	
Asian	2 (2)
Black or African American	8 (8)
White	88 (88)
Unknown	1 (1)
Other	1 (1)

CT, computed tomography; ELUCIDATE, Enabling Lung Cancer Identification Using Folate Receptor Targeting; NSCLC, non-small cell lung cancer.

**TABLE 2:** Characteristics of Occult Lesions Identified by Intraoperative Molecular Imaging With Pafolacianine

Variables	All Occult Lesions, n or n (%)	Malignant Occult Lesions, n or n (%)

Sample size		
Lesions	29	10
Participants	23	8
Size on pathologic examination, cm		
≥1 to ≤2.0	4 (14)	4 (40)
<1.0	12 (41)	6 (60)
No measurable lesion	13 (45)	0 (0)
Histopathologic type		
NSCLC	7 (24)	7 (70)
Metastasis from nonpulmonary location (chordoma)	3 (10)	3 (30)
Adenocarcinoma in situ	1 (3)	1 (10)
Benign lesion	18 (62)	0 (0)
Location		
Within same lobe as primary lesion	17 (59)	6 (60)
Within a different lobe from the primary lesion	12 (41)	4 (40)
Any previous lung cancer history		
Yes	7 (24)	3 (37)
No	22 (76)	5 (63)
Any previous nonlung cancer history		
Yes	19 (65)	4 (50)
No	10 (35)	4 (50)

NSCLC, non-small cell lung cancer.

**TABLE 3:** Case Summary of 23 Participants With 29 Occult Lesions Identified by Intraoperative Molecular Imaging

ID	Age, y	Sex	Other Previous Cancer Hx	Previous Lung Cancer Hx	Size, cm	Location	Primary Lesion Histologic Type	Resection Performed	Size, cm	Location	Synchronous Lesion Histologic Type	Resection Performed

1	64	F	No	Yes	0.9	RLL	Parenchyma	Lobe	0.6	RLL	Parenchyma	Lobe
2	58	M	Yes	No	2.2	RUL	Adeno	Wedge	NM NM	RLL RLL	Parenchyma Parenchyma	Wedge Wedge
3	72	F	No	No	1.0	RUL	Adeno	Wedge	NM	RUL	Parenchyma	Wedge
4	78	F	Yes	Yes	4.2	RUL	Adeno	Lobe	2.0	RLL	Adeno in situ	Wedge
5	63	M	No	No	3.4	LUL	Adeno	Wedge	NM NM	LLL LUL	Parenchyma Parenchyma	Wedge Wedge
6	47	M	Yes	No	1.4	LLL	Met (chordoma)	Wedge	0.5	LLL	Chordoma	Wedge
									0.6	LLL	Chordoma	Wedge
									0.1	LLL	Parenchyma	Wedge
									0.3	LUL	Chordoma	Wedge
7	83	F	No	No	2.0	LLL	Adeno	Lobe	2.0	LUL	Adeno	Wedge
8	62	F	No	No	0.8	RUL	Adeno	Lobe	1.1	RUL	Adeno	Lobe
9	60	F	No	Yes	0.9	LUL	Adeno	Wedge	0.5	LUL	Adeno	Wedge
10	61	M	Yes	No	1.8	RLL	Solid fibrous tumor	Wedge	NM	RUL	Parenchyma	Wedge
11	68	F	Yes	No	2.5	LUL	Atypical carcinoid	segment	0.3	LUL	Adeno	segment
12	69	F	Yes	No	0.7	RLL	Met (melanoma)	Wedge	0.2	RML	Granuloma	Wedge
13	71	F	No	No	1.4	LUL	Adeno	Wedge	0.5	LLL	Organizing pneumonia	Wedge
14	79	M	Yes	No	1.5	LLL	Met (prostate)	Wedge	NM	LUL	Parenchyma	Wedge
15	72	F	Yes	No	0.9	LUL	Adeno	Wedge	NM	LUL	Parenchyma	Wedge
16	77	M	Yes	Yes	1.1	RUL	Met (pancreatic)	Wedge	NM	RUL	Parenchyma	Wedge
17	66	F	No	No	1.1	LLL	Adeno	Wedge	0.2	LUL	Adeno	Wedge
18	68	F	No	No	1.2	LLL	Adeno	Wedge	0.2	LLL	Granuloma	Wedge
19	71	F	Yes	No	1.7	RUL	Adeno	Wedge	NM	RUL	Parenchyma	Wedge
20	50	M	Yes	Yes	1.0	LUL	Met (colorectal)	Wedge	NM NM	LUL LLL	Parenchyma Parenchyma	Wedge Wedge
21	54	M	Yes	Yes	1.0	RUL	Met (colorectal)	Wedge	NM	RUL	Parenchyma	Wedge
22	61	F	No	No	1.0	RUL	Adeno	Lobe	1.0	RUL	Adeno	Lobe
23	63	F	Yes	Yes	NM	RUL	Parenchyma	Wedge	0.45	RUL	Adeno	Wedge

Adeno, adenocarcinoma; F, female; Hx, history; ID, identification; LLL, left lower lobe; LUL, left upper lobe; M, male; Met, metastasis; NM, no measurement (no lesion) on final pathologic examination; RLL, right lower lobe; RUL, right upper lobe.

**TABLE 4:** Characteristics of 23 Participants With Malignant Occult Non-Small Cell Lung Cancer Lesions Identified by Intraoperative Molecular Imaging

Characteristics	Participants With Malignant Occult Lesion(s)	Participants With Benign Occult Lesion(s)

Number of patients	8	14
Sex		
Female	8	7
Male	0	7
Age, y		
Mean (SD)	67.6 (± 8.5)	66.4 (± 8.4)
Median [IQR]	64.5 [60–83]	68.5 [50–79]
Pack-year smoking history, y		
Mean (SD)	29.5 (± 15.1)	23.9 (± 18.4)
Median [IQR]	33.5 [0–45]	25.5 [0–50]
Size of largest primary lesion		
Mean (SD)	17.9 (± 12.4)	14.1 (± 7.1)
Median [IQR]	11 [8–42]	11.5 [7–34]

IQR, interquartile range.

## References

[1] Predina JD, Fedor D, Newton AD, Intraoperative molecular imaging: the surgical oncologist’s north star. Ann Surg. 2017;266:e42–e44. 10.1097/SLA.0000000000002247 28837055 PMC11037005

[2] Karsalia R, Cheng NH, Teng CW, Cho SS, Harmsen S, Lee JYK. Second window ICG predicts postoperative MRI gadolinium enhancement in high grade gliomas and brain metastases. Neurosurg Focus Video. 2022;6:V8. 10.3171/2021.10.FOCVID21204 PMC9555347 36284582

[3] Tanyi JL, Randall LM, Chambers SK, A phase III study of pafolacianine injection (OTL38) for intraoperative imaging of folate receptor-positive ovarian cancer (study 006). J Clin Oncol. 2023;41:276–284. 10.1200/jco.22.00291 36070540 PMC12684809

[4] Predina JD, Newton A, Corbett C, Localization of pulmonary ground-glass opacities with folate receptor-targeted intraoperative molecular imaging. J Thorac Oncol. 2018;13:1028–1036. 10.1016/j.jtho.2018.03.023 29626619 PMC6015787

[5] de Wit JG, Vonk J, Voskuil FJ, EGFR-targeted fluorescence molecular imaging for intraoperative margin assessment in oral cancer patients: a phase II trial. Nat Commun. 2023;14:4952. 10.1038/s41467-023-40324-8 37587149 PMC10432510

[6] Sarkaria IS, Martin LW, Rice DC, Pafolacianine for intraoperative molecular imaging of cancer in the lung: the ELUCIDATE trial. J Thorac Cardiovasc Surg. 2023;166:e468–e478. 10.1016/j.jtcvs.2023.02.025 37019717 PMC12507096

[7] Gangadharan S, Sarkaria IN, Rice D, Multiinstitutional phase 2 clinical trial of intraoperative molecular imaging of lung cancer. Ann Thorac Surg. 2021;112:1150–1159. 10.1016/j.athoracsur.2020.09.037 33221195 PMC10985531

[8] Suzuki K Whack-a-mole strategy for multifocal ground glass opacities of the lung. J Thorac Dis. 2017;9(suppl 3):S201–S207. 10.21037/jtd.2017.04.03 28446985 PMC5392542

[9] Kim YW, Lee CT. Optimal management of pulmonary ground-glass opacity nodules. Transl Lung Cancer Res. 2019;8(suppl 4):S418–S424. 10.21037/tlcr.2019.10.24 32038928 PMC6987366

[10] Ramirez RA, Cass AS, Das S, A multidisciplinary approach to the work up and management of pulmonary carcinoid tumors and DIPNECH: a narrative review. Transl Lung Cancer Res. 2022;11:2567–2587. 10.21037/tlcr-22-415 36636417 PMC9830261

[11] Predina JD, Newton AD, Keating J, A phase I clinical trial of targeted intraoperative molecular imaging for pulmonary adenocarcinomas. Ann Thorac Surg. 2018;105:901–908. 10.1016/j.athoracsur.2017.08.062 29397932 PMC10959252

[12] Baker N, Sarkaria I, Alicuben E, Ajabshir N, Levy R. First reported real-world use of pafolacianine and intraoperative molecular imaging for nodule localization and occult tumor detection in malignant lung lesions: results of a single institution pilot. Paper presented at: One hundred fourth American Association for Thoracic Surgery Annual Meeting; April 28, 2024; Toronto, Ontario, Canada.

[13] Cerfolio RJ, Bryant AS. Is palpation of the nonresected pulmonary lobe(s) required for pateints with non-small cell lung cancer? A prospective study. J Thorac Cardiovasc Surg. 2008;135:261–268. 10.1016/j.jtcvs.2007.08.062 18242247

[14] Azari F, Kennedy G, Bernstein E, Evaluation of OTL38-generated tumor-to-background ratio in intraoperative molecular imaging-guided lung cancer resections. Mol Imaging Biol. 2023;25:85–96. 10.1007/s11307-021-01618-9 34101106 PMC8651846

